# Extended Regression Analysis for Debye–Einstein Models Describing Low Temperature Heat Capacity Data of Solids

**DOI:** 10.3390/e26060452

**Published:** 2024-05-26

**Authors:** Ernst Gamsjäger, Manfred Wiessner

**Affiliations:** 1 Institute of Mechanics, Montanuniversität Leoben, Franz-Josef-Str. 18, 8700 Leoben, Austria; 2 Anton Paar GmbH, Anton-Paar-Str. 20, 8054 Graz, Austria; manfred.wiessner@anton-paar.com

**Keywords:** thermodynamic functions, Bayesian framework, probability density distribution, regression analysis, Markov chain Monte Carlo (MCMC)

## Abstract

Heat capacity data of many crystalline solids can be described in a physically sound manner by Debye–Einstein integrals in the temperature range from 0K to 300K. The parameters of the Debye–Einstein approach are either obtained by a Markov chain Monte Carlo (MCMC) global optimization method or by a Levenberg–Marquardt (LM) local optimization routine. In the case of the MCMC approach the model parameters and the coefficients of a function describing the residuals of the measurement points are simultaneously optimized. Thereby, the Bayesian credible interval for the heat capacity function is obtained. Although both regression tools (LM and MCMC) are completely different approaches, not only the values of the Debye–Einstein parameters, but also their standard errors appear to be similar. The calculated model parameters and their associated standard errors are then used to derive the enthalpy, entropy and Gibbs energy as functions of temperature. By direct insertion of the MCMC parameters of all $4\cdot 10^{5}$ computer runs the distributions of the integral quantities enthalpy, entropy and Gibbs energy are determined.

## 1. Introduction

Some sets of model parameters used to fit low temperature heat capacity measurements are purely empirical (see, e.g., [1,2,3,4]), other parameters are at least partly motivated by theory, e.g., following the Debye–Einstein approach [5,6,7,8,9,10,11,12]. A Debye–Einstein integral to describe heat capacities of several compounds is introduced by Kelley and King [5]. They proposed a theory-based heat capacity function that contains two parts, where a *p*-atomic isotropic crystal consists of one Debye term and (p−1) Einstein terms. The value *p* equals the number of atoms in a molecule or else the number of atoms in the simplest chemical formula that may be written to represent the composition. A modification of this heat capacity function can be found in Wu et al. [10]. It is demonstrated in [12] that the temperature-dependent standard molar heat capacities ($C_{p,m}^{o}\left(T\right)$) data for many crystalline solids can be described by means of these Debye–Einstein integrals over the low temperature range (0–300 K).

The Debye–Einstein regression analysis only requires a small number of thermodynamically motivated fitting parameters. Unlike using fitting polynomials or splines, it is possible to extrapolate the Debye–Einstein model to zero Kelvin, even if experimental $C_{p,m}^{o}$ data are lacking for ultra-low temperatures, e.g., below 50K (details can be found in [9]). Compared to various models (e.g., polynomial fits) that require a large number of fit parameters over the range of 0–300 K, the Debye–Einstein approach offers the advantage of easy tabulation of a few (four to six) fit parameters in a systematic manner for different crystalline solids. Moreover, models with a high number of fit parameters often encounter the issue of overfitting, which becomes evident when the uncertainties associated with these fit parameters are comparable to or even exceed the magnitude of the parameters themselves. In general, $C_{p,m}^{o}$ values increase strongly, ranging from very small values close to 0K to increasingly high values at T=298.15K. In addition, the $C_{p,m}^{o}$ values increase with the number of atoms in the formula unit. This possible wide range of variation indicates that the standard errors associated with the residuals also change with temperature.

Thermodynamic data are often fitted by means of various simply empirical or thermodynamically motivated models, where the uncertainties of the fit parameters are not provided (see, e.g., [13]). Frequently, the correlation of the fit parameters is missing too. However, uncertainty estimation is now the focus of recently published papers, e.g., in [14], where experimental data and atomistic experiments are included in the model development. In general, thermodynamic modeling relies on the significance of the experimental data, their availability and on the adequacy of the model functions. In this context, it is pointed out in Honarmandi et al. [15] that uncertainty quantification of phase diagrams is of paramount importance for decision making in materials design. Paulson et al. [16] state that uncertainty quantification in combination with CALPHAD is not yet widely adopted, and present uncertainty quantification of the properties from CALPHAD modeling and make their program codes available. Uncertainty quantification in thermodynamic modeling follows either from classical (frequentist) statistics (e.g., [17]) or when Bayesian statistics (e.g., [15,16,18]) are applied. One advantage of Bayesian interference is that prior knowledge can be introduced into the calculations updated by the likelihood function, which is influenced by new measurements in order to obtain the posterior probability. A direct calculation of the posterior probability in the *n*-dimensional problem set (*n* parameters are sought) is almost impossible. However, by means of modern data sampling techniques using the Markov chain Monte Carlo (MCMC) method the posterior probability distribution is approximated (see, e.g., Vrugt and Ter Braak [19]). The MCMC method leads to a comparatively fast convergence of high-dimensional problems and is, therefore, becoming increasingly popular.

In this work, the estimated distribution of the parameters of the Debye–Einstein fit are obtained from the local Levenberg–Marquardt least squares minimization and from the global MCMC regression tool. In the case of MCMC regression the standard errors of the experimental heat capacities are described as a function with parameters derived from the residuals (i.e., the deviations of the measured values to the calculated values at the measured heat capacities). In this way, unknown systematic errors are indirectly introduced into the error calculations [20]. Unknown systematic errors due to a specific model occur since all models are only a simplification of reality [21]. The parameters of the function describing the standard errors are simultaneously optimized with the parameters of the Debye–Einstein heat capacity function. Thereby, the Bayesian credible interval is calculated for the temperature-dependent heat capacities $C_{p,m}^{o}\left(T\right)$. The molar enthalpy $H_{m}^{o}\left(T\right)$, molar entropy $S_{m}^{o}\left(T\right)$ and the derived function $\left(-G_{m}^{o}/T\right)\left(T\right)$ are derived from the $C_{p,m}^{o}\left(T\right)$ values.

The standard errors of the parameters are calculated by means of the classical least squares method and the best estimates of the fit parameters are obtained by using the global MCMC method. It is shown that the estimated distributions of the parameters calculated from the MCMC regression fit well to the standard errors which follow from the classical least squares method.

## 2. Literature Data and Theory

Calorimetric measurements of heat capacity data for various crystalline solids have been extensively reported in the literature (see e.g., [2,10,22,23,24,25,26]). These measurements cover a temperature range from ultra-low temperatures, such as 2K, up to 300K or slightly higher temperatures. The heat capacity data are commonly obtained using the relaxation method, e.g., provided by Quantum Design Physical Property Measurement Systems, San Diego, CA, USA [27]. The uncertainties associated with the measurements, including instrumental errors and statistical fluctuations, were rigorously evaluated and reported in [28] and usually come along with a relative uncertainty $\sigma_{C_{p}}/C_{P}=5\cdot 10^{-3}$ for T<50K and $\sigma_{C_{p}}/C_{P}=3\cdot 10^{-3}$ for T>50K.

In the following, the Debye–Einstein integral fit of heat capacity data of ${\mathrm{SrMoO}}_{4}$ is presented, which is described by six parameters only in the low temperature range (0–320 K). The underlying experimental data are published in Morishita and Houshiyama [26].

### 2.1. Debye–Einstein Integral

In our study, we employ the Debye–Einstein integral fit (Equation (1)) proposed by Wu et al. [10] and extend its application to the entire measurement range (see also Gamsjäger and Wiessner [12] and Ogris and Gamsjäger [29]):

(1) $$C_{p,m}^{o}=mD\left(T_{D}/T\right)+n_{1}E_{1}\left(T_{E1}/T\right)+n_{2}E_{2}\left(T_{E2}/T\right)$$

where the Debye integral $D(T_{D}/T)$ is given by:

(2) $$D\left(T_{D}/T\right)=9R{\left(\frac{T}{T_{D}}\right)}^{3}\int_{0}^{T_{D}/T}\frac{y^{4}\exp\left(y\right)}{{\left[\exp\left(y\right)-1\right]}^{2}}\;dy$$

and the two Einstein terms $E_{1}\left(T_{E1}/T\right)$ and $E_{2}\left(T_{E2}/T\right)$ are given by:

(3) $$E_{i}\left(T_{Ei}/T\right)=3R\frac{{\left(\frac{T_{Ei}}{T}\right)}^{2}\cdot \exp\left(T_{Ei}/T\right)}{{\left[\exp\left(T_{Ei}/T\right)-1\right]}^{2}}$$

These equations involve the following fit parameters: the Debye temperature $T_{D}$, the Einstein temperatures $T_{Ei}$ with i=1 or i=2, and the prefactors *m*, $n_{1}$ and $n_{2}$. According to theory, the sum of the prefactors are equal to the number of atoms in the formula unit as can be found in [5]. Thus, the low temperature $C_{p,m}^{o}$ measurements are described by the Debye–Einstein model. Starting from the temperature-dependent standard molar heat capacity $C_{p,m}^{o}\left(T\right)$, the molar enthalpy $H_{m}^{o}\left(T\right)$ and the molar entropy $S_{m}^{o}\left(T\right)$ are obtained by integration as follows:

(4) $$H_{m}^{o}\left(T\right)=\int_{0}^{T}C_{p,m}^{o}\left(\bar{T}\right)\;d\bar{T}$$

and

(5) $$S_{m}^{o}\left(T\right)=\int_{0}^{T}\frac{C_{p,m}^{o}\left(\bar{T}\right)}{\bar{T}}\;d\bar{T}$$

It is worth noting that the Debye–Einstein integral approach allows for extrapolation to absolute zero in the case that ultra-low temperature data are missing; e.g., data are compiled for T>50K only (see, e.g., [9,29]). The prefactors *m*, $n_{1}$ and $n_{2}$ describe the weight of the Debye integral and the Einstein terms, respectively, where the sum is not a priori fixed in the fitting algorithm, but appears to be close to the number of atoms in the formula unit of the investigated compound as it should be from a theoretical point of view (see also [12]). This is an indication that the fit parameters used in the Debye–Einstein integral are not only of an empirical nature, but are relevant with respect to the theory behind. In addition to the Levenberg–Marquardt least squares calculations for finding the optimized parameters of the Debye–Einstein integral, the optimal values of these fit parameters within their distributions are estimated by means of the MCMC method within a Bayesian framework.

### 2.2. Bayesian Framework and MCMC Regression

Unlike traditional optimization methods that rely solely on minimizing the sum of least squares of the residuals, the Bayesian approach offers a probabilistic framework for incorporating prior knowledge, estimating model parameters and quantifying uncertainties. Bayes’ theorem is derived from the product rule of conditional probabilities (see, e.g., [30]). The conditional probability P(B|A) represents the probability of event *B* given that event *A* is true. Applying Bayes’ theorem for our case, the posterior probability $P\left(\vec{\xi }|D,H\right)$ follows from the following Equation (6):

(6) $$P\left(\vec{\xi }|D,H\right)=\frac{P\left(D|\vec{\xi },H\right)\cdot P\left(\vec{\xi }|H\right)}{P\left(D|H\right)}$$

The prior probability distribution $P\left(\vec{\xi }|H\right)$ incorporates any existing prior knowledge about the vector of the parameters $\vec{\xi }$ within the hypothesis space *H*.

The prior distribution is updated with new data *D* by using the likelihood function $P\left(D|\vec{\xi },H\right)$. The likelihood function evaluates how close the fit function containing the parameters $\vec{\xi }$ approaches the experimental data. The product of the likelihood function $P\left(D|\vec{\xi },H\right)$ and the prior probability $P\left(\vec{\xi }|H\right)$ is then normalized by the evidence $P\left(D|H\right)$, resulting in the posterior probability $P\left(\vec{\xi }|D,H\right)$.

In our investigation, the vector $\vec{\xi }$ consists of the six parameters of the Debye–Einstein integral, i.e., the prefactors *m*, $n_{1}$, $n_{2}$, the Debye temperature $T_{D}$ and the Einstein temperatures $T_{E1}$ and $T_{E2}$, and the parameters $s_{0}$ and $s_{1}$ of the function of the standard errors. It is assumed that the hypothesis space *H* remains constant, which implies that the distribution $P\left(D|H\right)$, commonly referred to as evidence, also remains constant. In our case, boundaries are imposed on the hypothesis space *H*, since the model parameters have to be positive; $m\in R^{+}$, $n_{1}\in R^{+}$, $n_{2}\in R^{+}$, $T_{D}\in R^{+}$, $T_{E1}\in R^{+}$, $T_{E2}\in R^{+}$.

The Bayesian equation is often transformed into its logarithmic form due to numeric advantages:

(7) $$\ln P\left(\vec{\xi }|D,H\right)=\ln P\left(D|\vec{\xi },H\right)+\ln P\left(\vec{\xi }|H\right)-\ln P\left(D|H\right)$$

For the likelihood function in Equation (6) or Equation (7), it is commonly used to employ the Gaussian distribution $Gauss(y_{i},y_{c,i},\sigma_{i})$ to calculate the probability for each measurement point.

(8) $$Gauss\left(y_{i},y_{c,i},\sigma_{i}\right)=\frac{1}{\sqrt{2\pi }}\cdot \frac{1}{\sigma_{i}}\exp\left(-\frac{{\left[y_{i}-y_{c,i}\right]}^{2}}{2\sigma_{i}^{2}}\right)$$

Furthermore, in a “naive” manner, it is commonly assumed that the residuals, i.e., the measured heat capacities minus the calculated heat capacity values $\left(y_{i}-y_{c,i}\right)$ for all *i* data points, are independent of each other. Therefore, the likelihood function is obtained by multiplying the individual probabilities of each Gaussian distribution.

In this Bayesian framework, the standard errors of the residuals are estimated. To reduce the number of fit parameters to a manageable level, we propose to apply a simple function with the parameters ${\vec{\xi }}_{2}$ that describes these standard errors. The standard errors are influenced by the uncertainties in the measurements and unknown errors brought in by the model. The logarithmic posterior distribution that accounts for the function of the standard errors is written as

(9) $$\begin{array}{c} \ln P\left(\left\{\;{\vec{\xi }}_{1},{\vec{\xi }}_{2}\right\}|\left\{\left(x_{i},y_{i}\right)\right\},\;H\right)=-\ln P\left(\{\left(x_{i},y_{i}\right)\}\;|\;H\right)-\frac{n}{2}\ln\left(2\pi \right)\\ \;\;\;\;\;\;\;-\sum_{i=1}^{n}\left\{\ln\left[\sigma_{c,i}\left(y_{i},{\vec{\xi }}_{2}\right)\right]+\frac{{\left[y_{i}-y_{c,i}\left({\vec{\xi }}_{1}\right)\right]}^{2}}{2\sigma_{c,i}^{2}\left(y_{i},{\vec{\xi }}_{2}\right)}\right\}+\ln\left(P\left(\{\;{\vec{\xi }}_{1},{\vec{\xi }}_{2}\;\}\right)\;|\;H\right) \end{array}$$

Assuming a flat prior (no prior knowledge), the posterior distribution simplifies to Equation (10):

(10) $$\begin{array}{c} \ln P\left(\left\{\;{\vec{\xi }}_{1},{\vec{\xi }}_{2}\right\}|\left\{\left(x_{i},y_{i}\right)\right\},\;H\right)=-\ln\left[P\left(\{\left(x_{i},y_{i}\right)\}\;|\;H\right)\right]-\frac{n}{2}\ln\left(2\pi \right)\\ \;\;\;\;\;\;\;\;\;\;\;\;\;\;\;-\sum_{i=1}^{n}\left\{\ln\left(\sigma_{c,i}\left(y_{i},{\vec{\xi }}_{2}\right)\right)+\frac{{\left(y_{i}-y_{c,i}\left({\vec{\xi }}_{1}\right)\right)}^{2}}{2\sigma_{c,i}^{2}\left(y_{i},{\vec{\xi }}_{2}\right)}\right\} \end{array}$$

The Markov chain Monte Carlo (MCMC) sampling method can be used effectively within the Bayesian framework. MCMC allows us to explore the parameter space with the aim to eventually converge to the joint posterior probability. Thereby, robust estimates of the parameters are possible and their associated standard errors are obtained from the posterior probability density distributions.

In our study, we employ an advanced version of the Metropolis–Hastings algorithm, known as the Differential Evolution Adaptive Metropolis (DREAM) algorithm, which was initially developed by Braak [31] and further enhanced by Vrugt and Ter Braak [19]. The DREAM algorithm is specifically designed for Bayesian optimization and incorporates multiple chains with differential evolution and adaptive Metropolis–Hastings steps. This MCMC approach substantially enhances the exploration of the parameter space by dynamically adjusting the step sizes, leading to improved convergence and efficiency in the optimization process. The relative frequencies of parameter occurrences within the parameter range directly correspond to their probability density distribution. The standard errors of the parameters equal the standard deviations of these parameters and are calculated by considering all values from all Markov chains. These MCMC-based standard errors can be compared with the standard errors obtained from the error propagation rule from classical statistics.

In the following, the correlations between the parameters are estimated. The covariance between two parameters can be calculated using the following formula:

(11) $$\mathrm{cov}\left(A,B\right)=\frac{1}{N}\sum_{i=1}^{N}\left(A_{i}-\bar{A}\right)\left(B_{i}-\bar{B}\right)$$

Here, *N* represents the total number of samples or observations of all Markov chains, $A_{i}$ and $B_{i}$ are the values of parameters *A* and *B* for the *i*th element of the Markov chains, and $\bar{A}$ and $\bar{B}$ denote the mean values of the parameters *A* and *B* of all entries in the Markov chains, respectively.

The correlation coefficient, denoted as *r*, is defined as:

(12) $$r=\frac{\mathrm{cov}(A,B)}{\sigma_{A}\sigma_{B}}$$

In this equation, $\sigma_{A}$ and $\sigma_{B}$ represent the standard errors of parameters *A* and *B*, respectively.

## 3. Results and Discussion

The molar heat capacities $C_{p,m}^{o}$ of ${\mathrm{SrMoO}}_{4}$ have been measured over a temperature range from 2K to 320K by Morishita and Houshiyama [26] using a relaxation method instrument. As an example, these data, i.e., 81 $C_{p,m}^{o}\left(T\right)$ data pairs, are evaluated in this work by means of the Debye–Einstein approach using both methods, least squares minimization and Bayesian statistics, with the help of Monte Carlo Markov chains (MCMC). Regression by the latter method is based on the DREAM algorithm. For the analyses, we used 10 chains, each consisting of $5\cdot 10^{4}$ iterations, with the initial $1\cdot 10^{4}$ iterations per chain discarded as burn-in. This means that a total of $4\cdot 10^{5}$ parameter sets were available for analysis.

**Figure 1:**
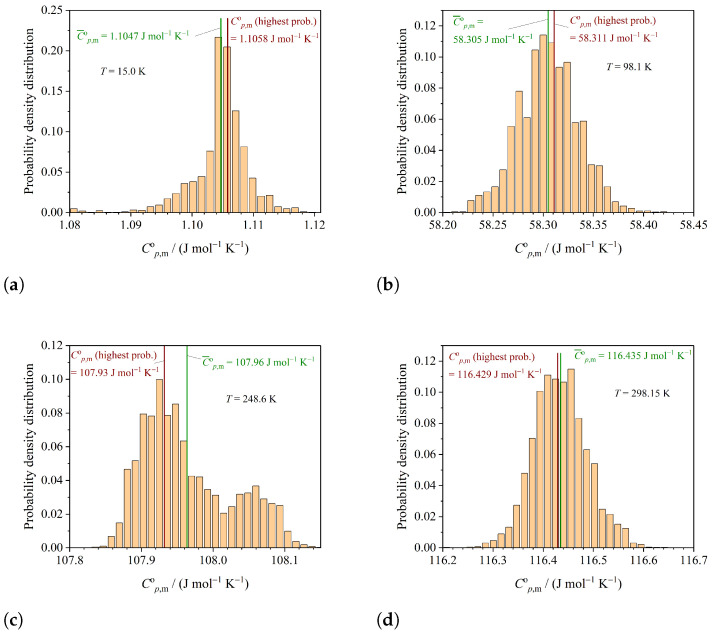
Probability densities of the heat capacities $C_{p,m}^{o}$ for ${\mathrm{SrMoO}}_{4}$ at selected temperatures *T*. (**a**) Probability density of $C_{p,m}^{o}$ at T=15.0K. (**b**) Probability density of $C_{p,m}^{o}$ at T=98.1K. (**c**) Probability density of $C_{p,m}^{o}$ at T=199.9K. (**d**) Probability density of $C_{p,m}^{o}$ at T=199.9K.

The probability density distributions of the simulated heat capacities follow from the MCMC approach by fitting the experimental heat capacities. These probability density distributions are presented at selected temperatures. It is worth noting that these distributions, that follow from the six-parameter Debye–Einstein integral, can be extrapolated to lower temperatures in the case of lacking experimental data. The probability density distribution of $C_{p,m}^{o}$ at T=15.0K is presented in Figure 1a, the probability density distribution of $C_{p,m}^{o}$ at T=98.1K is shown in Figure 1b, and the probability density distributions at T=248.6K and T=298.15K can be seen in Figure 1c,d, respectively. The probability density distribution of $C_{p,m}^{o}$ at T=248.6K (Figure 1c) exhibits two distinct maxima. It can be speculated that these two maxima occur due to correlation between the parameters induced by the non-linear behavior of the Debye–Einstein approach. In such a case, the Bayesian approach results in more realistic error estimations compared to classical error propagation analysis. In the case of many local minima, global regression analysis is recommended to reliably estimate the error of the regression, as is shown for an example from X-ray diffraction data analysis in [32].

**Figure 2:**
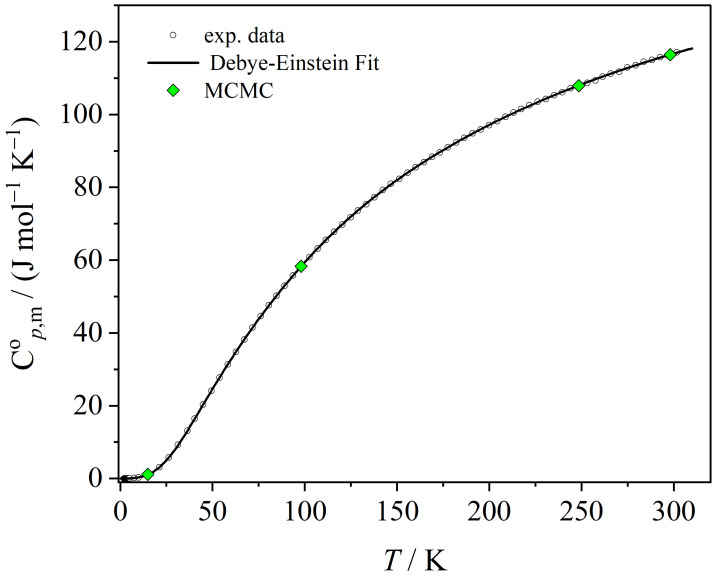
Experimental $C_{p,m}^{o}$ values from [26] versus *T* approximated by the 6p-Debye–Einstein integral fit; the mean values of the $C_{p,m}^{o}$ values obtained by the MCMC-method are also shown.

The experimental data for $C_{p,m}^{o}$ of ${\mathrm{SrMoO}}_{4}$, taken from [26], are plotted versus *T* in Figure 2. The solid line in Figure 2 corresponds to the least squares Levenberg–Marquardt fit of the six-parameter Debye–Einstein integral, computed by means of Origin2022b [33]. The mean values of the $C_{p,m}^{o}$ probability distribution densities are also plotted in Figure 2. Both, the classical least squares six-parameter (6p)-Debye–Einstein fit and the MCMC calculation mimic the experimental data almost perfectly well.

The six parameters of the Debye–Einstein fit and their standard errors are calculated for both methods—the classical least squares (LSQ) method and the MCMC approach—and are listed in Table 1. In the case of the MCMC approach, the mean value of the probability density distribution and the highest probability is calculated, as well as the standard errors which follow from the standard deviations of all values calculated in the MCMC approach. The parameters obtained by both, completely different, regression approaches result in values for the parameters that are very close and even the estimated standard errors are similar.

**Figure 3:**
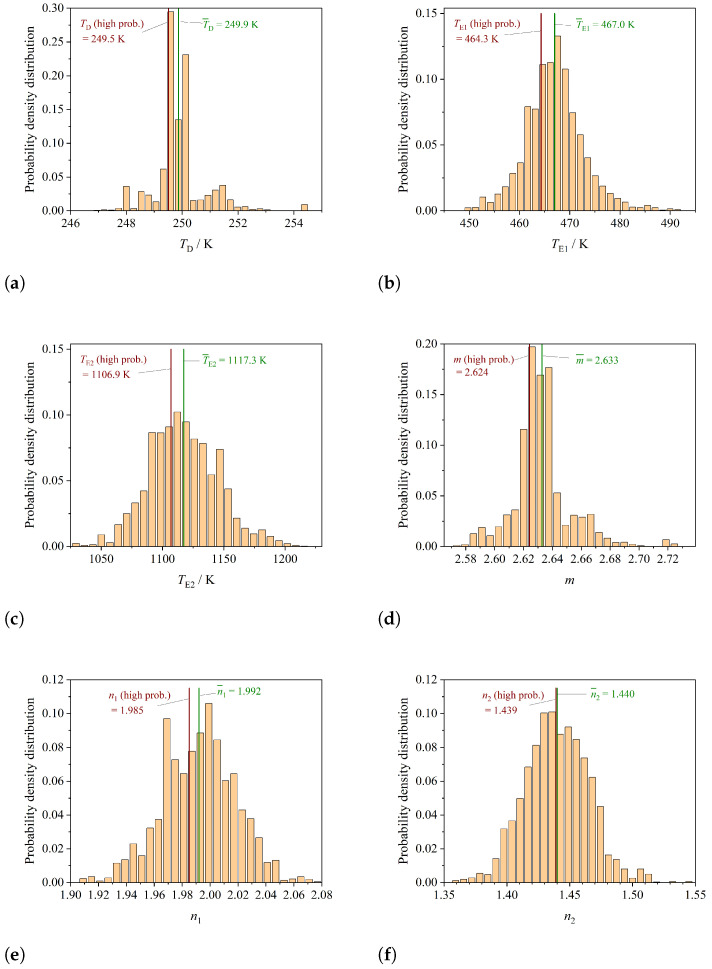
Probability densities of the 6 parameters of the Debye–Einstein integral. (**a**) Probability density of the Debye temperature $T_{D}$. (**b**) Probability density of the Einstein temperature $T_{E1}$. (**c**) Probability density of the Einstein temperature $T_{E2}$. (**d**) Probability density of the factor *m*. (**e**) Probability density of the factor $n_{1}$. (**f**) Probability density of the factor $n_{2}$.

The probability density distributions of the model parameters are calculated, and presented in Figure 3. The probability density distribution for the Debye temperature $T_{D}$ is presented in Figure 3a, for the Einstein temperatures $T_{E1}$ in Figure 3b and $T_{E2}$ in Figure 3c, respectively. The probability density distributions of the prefactors *m*, $n_{1}$ and $n_{2}$ are shown in Figure 3d, Figure 3e and Figure 3f, respectively.

In addition, the parameters of the Debye–Einstein model function, as provided in Table 1, and the parameters of the function describing the errors of the heat capacities are simultaneously optimized.

### 3.1. Estimating the Uncertainties of Each Measurement Point

Our objective is to identify a function as simply as possible to approximate the standard errors of the data points investigated, where the experimental data are provided in [26]. This function must obey the following two criteria:

- The temperature dependency of the residuals should adequately describe the temperature dependency of the standard errors and vice versa, e.g., as the residuals increase with increasing heat capacities, the standard errors should also increase with increasing heat capacities.
- The correlations between the parameters in the function for the standard errors should not be excessively high (e.g., above 90 percent), as this indicates the potential for using a simpler standard error function without significant data loss.

Since the distribution of the residuals is not known beforehand, the evaluation is carried out iteratively, and in case of failure, the entire analysis must be repeated by using another function for the standard errors. The following functions may be considered for describing the standard errors:

(13) $$s\left(C_{p,m}^{o}\right)=s_{0}$$

(14) $$s\left(C_{p,m}^{o}\right)=s_{0}+s_{1}\cdot \left|C_{p,m}^{o}\right|$$

(15) $$s\left(C_{p,m}^{o}\right)=s_{0}+s_{1}\cdot \sqrt{|C_{p,m}^{o}|}$$

**Figure 4:**
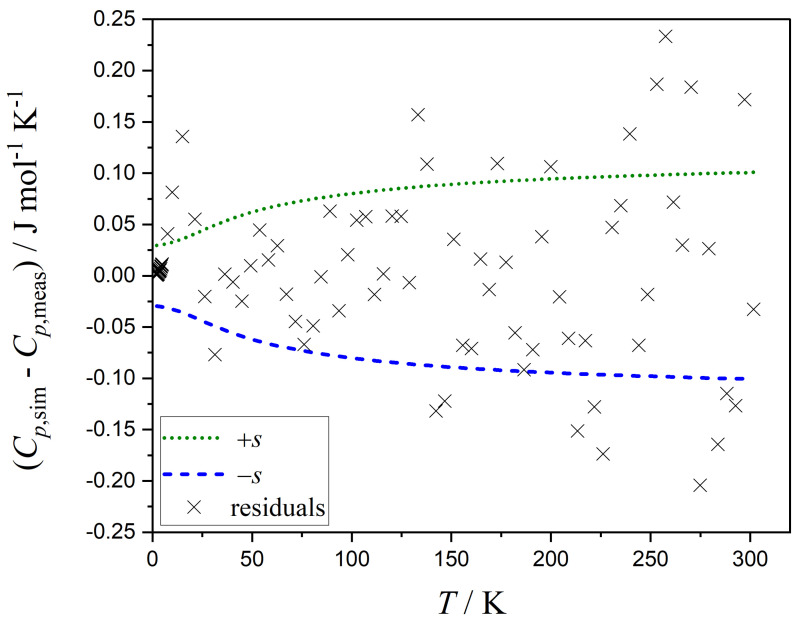
Residuals (difference between simulated and measured heat capacities, $C_{p,\mathrm{sim}}-C_{p,\mathrm{meas}}$) as a function of temperature *T*. The function *s* of the standard errors and this function mirrored at the abscissa, i.e., −s, are also plotted versus *T*.

It is worth noting that the function *s* must remain positive over the whole range of $C_{p,m}^{o}$. The simplest approach is to assign an equal, i.e., constant, standard error to all data points (Equation (13)). However, a better choice may consider the increase of the residuals with increasing heat capacities. A high correlation between $s_{0}$ and $s_{1}$ is observed when using a linearly increasing function (Equation (14)). When Equation (15) is used to describe the standard error function, a more realistic distribution of residuals is obtained. Figure 4 displays the residuals versus *T*. The residuals are calculated from the Markov chain containing the parameters with the highest probability. The function of the standard error *s* together with −s, i.e., the credible interval, is calculated from Equation (15) and plotted versus *T* in Figure 4.

The parameters $s_{0}$ and $s_{1}$ of the function describing the standard error of $C_{p,m}^{o}$ versus *T* are listed in Table 2.

### 3.2. Determining the Correlation between Parameters

In this section, the correlation between all parameters is determined using Equation (12). The resulting correlation matrix, which is symmetric, is presented in Table 3.

**Figure 5:**
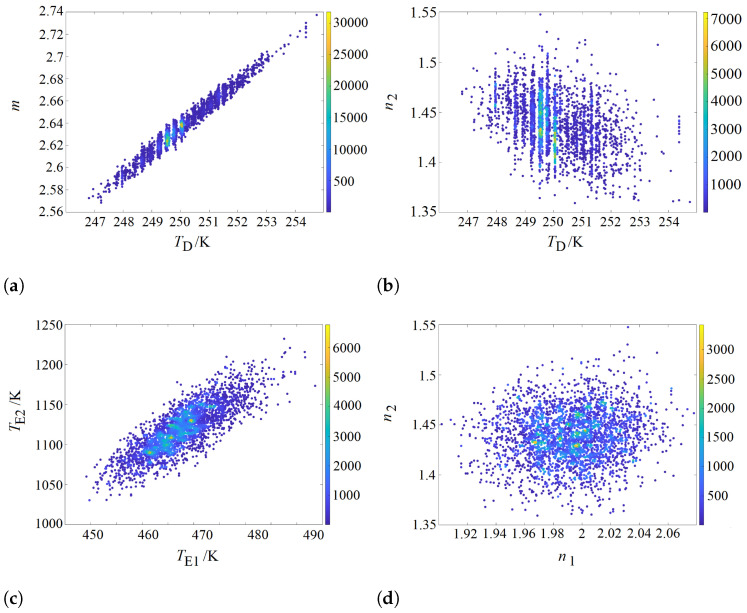
Color -coded scatter plots visualizing the correlation between certain parameters. (**a**) Correlation between $T_{D}$ and *m*. (**b**) Correlation between $T_{D}$ and $n_{2}$. (**c**) Correlation between $T_{E1}$ and $T_{E2}$. (**d**) Correlation between $n_{1}$ and $n_{2}$.

The values specified in the correlation matrix can be visualized by scatter plots (Figure 5) showing the correlation of two selected parameters. The points in these scatter plots are color-coded. The color of the points changes with the frequency of hits (axis on the right) in the range of the parameter space represented by the point.

As a representative example, the correlation between the Debye temperature $T_{D}$ and the prefactor *m* is illustrated in Figure 5a, which is very high and at 0.98 close to 1. These two parameters are almost linearly related. However, neither of these two parameters can be omitted, since the Debye integral has to have a certain weight *m* not known before the regression analysis. The parameters $T_{D}$ and $n_{2}$ are slightly anti-correlated, with a value of −0.31, as can be seen in Figure 5b. Whereas the Einstein temperatures are strongly correlated at 0.83, as shown in Figure 5c, the prefactors $n_{1}$ and $n_{2}$ are practically not correlated and the value of 0.09 results in a scatter plot which is symmetric to the abscissa (Figure 5d).

In this example, the condition number of the correlation matrix *r* is calculated to be 5700, which indicates a high value. This high condition number suggests that the equation system is poorly conditioned. Therefore, from this perspective, the use of a more complex model (e.g., incorporating additional Einstein terms) is not recommended.

**Figure 6:**
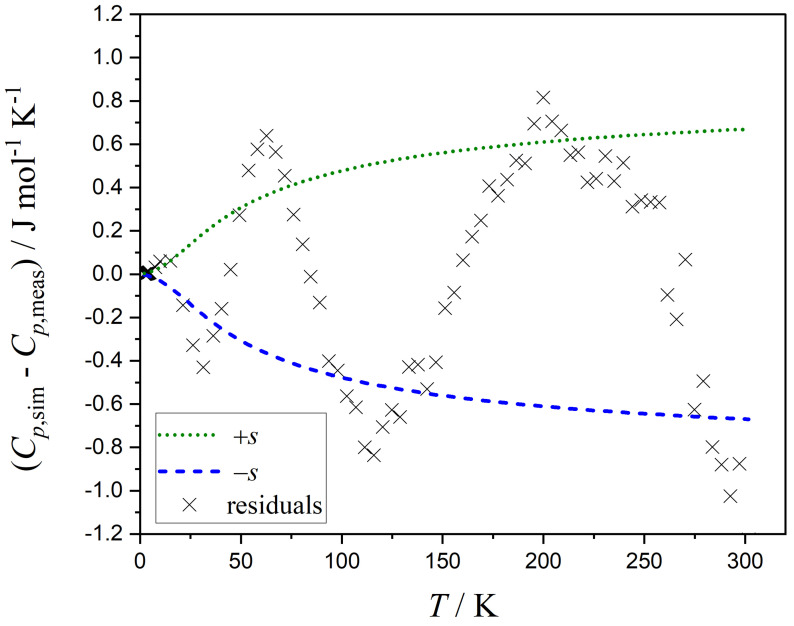
Residuals (difference between simulated and measured heat capacities, $C_{p,\mathrm{sim}}-C_{p,\mathrm{meas}}$) in the case of a simpler 4p-Debye–Einstein fit as a function of temperature *T*. The function *s* of the standard errors and this function mirrored at the abscissa, i.e., −s are also plotted versus *T*.

Based on the residuals analysis discussed in the section “Estimating the Uncertainties of Each Measurement Point”, it can be concluded that underfitting is not observed in the examined dataset. Moreover, the inclusion of additional Einstein terms would not lead to a substantial reduction in the residuals. The question may arise if a four-parameter (4p) Debye–Einstein integral with a Debye temperature $T_{D}$ and an Einstein temperature $T_{E}$ and their prefactors *m* and *n* suffices to describe $C_{p,m}^{o}\left(T\right)$ of ${\mathrm{SrMoO}}_{4}$ from 0K to 300K. Thus, the residuals following from a simpler four-parameter Debye–Einstein approach are calculated by the MCMC approach and presented in Figure 6.

Compared to the residuals of the 6p-Debye–Einstein fit, shown in Figure 4, the 4p-fit results in almost five times larger residuals (Figure 6), which are not randomly distributed for this compound. This means that the 6p-Debye–Einstein fit seems to be the better option for describing the heat capacities $C_{p,m}^{o}$ of ${\mathrm{SrMoO}}_{4}$ than the simpler 4p-fit. In addition, it is shown in [12] that the 6p-Debye–Einstein approach leads to a heat capacity description with small standard errors of the fit parameters for many compounds.

### 3.3. Thermodynamic Functions

The molar entropy $S_{m}^{o}\left(T\right)$ and molar enthalpy $H_{m}^{o}\left(T\right)$ can be determined by using Equations (16) and (17), i.e., integrating the simulated molar heat capacities $C_{p,m}^{o}\left(T\right)$ numerically.

(16) $$S_{m}^{o}\left(T\right)=\int_{0}^{T}\frac{C_{p,m}^{o}\left(\bar{T}\right)}{\bar{T}}d\bar{T}$$

(17) $$H_{m}^{o}\left(T\right)=\int_{0}^{T}C_{p,m}^{0}\left(\bar{T}\right)d\bar{T}$$

The derived function $-G_{m}^{o}/T$, with $G_{m}^{o}$ being the molar Gibbs energy, is obtained from:

(18) $$\frac{-G_{m}^{o}\left(T\right)}{T}=S_{m}^{o}\left(T\right)-\frac{H_{m}^{o}\left(T\right)}{T}$$

The values of the thermodynamic functions $S_{m}^{o}$, $H_{m}^{o}$ and $S_{m}^{o}\left(T\right)-H_{m}^{o}\left(T\right)/T$ of ${\mathrm{SrMoO}}_{4}$ at T=298.15K are presented in Table 4. The values are obtained from Levenberg–Marquardt least squares analysis (see also [12]). These values are compared to those of the highest probability calculated with the MCMC approach and to values from [26].

This approach allows for the evaluation of entropy as a function of temperature for each set of fit parameters obtained from the Monte Carlo Markov chains. The individual entropy profiles serve as the basis for generating histograms at specific temperatures.

**Figure 7:**
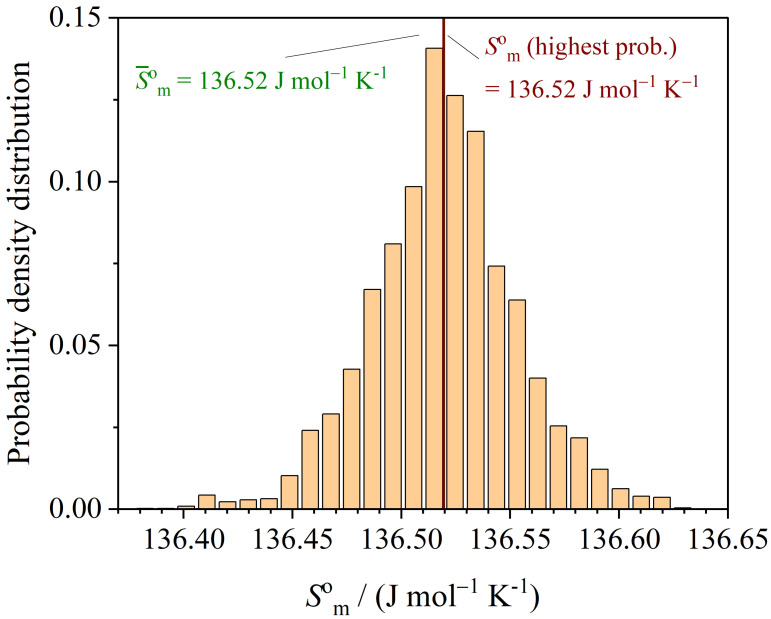
Probability density distribution of $S_{m}^{o}$ at T=298.15K.

The histogram (probability density distribution) of the molar entropy $S_{m}^{o}$ at T=298.15K is shown in Figure 7 providing insights into the distribution of entropy values. The mean entropy value is determined to be $136.5196\;J\;{\mathrm{mol}}^{-1}\;K^{-1}$. Additionally, the entropy value with the highest probability corresponds to $136.5195\;J\;{\mathrm{mol}}^{-1}\;K^{-1}$, representing the most likely entropy state of the system at the given temperature.

Furthermore, the standard deviation, a measure of the uncertainty of entropy values around the mean, can be calculated as $\Delta S_{m}^{o}=0.033\;J\;{\mathrm{mol}}^{-1}\;K^{-1}$.

**Figure 8:**
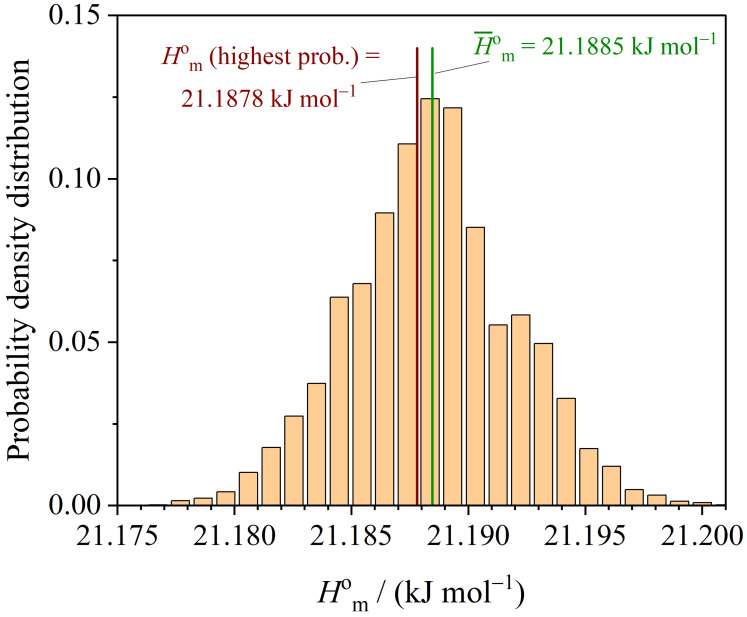
Probability density distribution of $H_{m}^{o}$ at T=298.15K.

The probability density distribution of the enthalpy $H_{m}^{o}$ at T=298.15K is shown in Figure 8.

**Figure 9:**
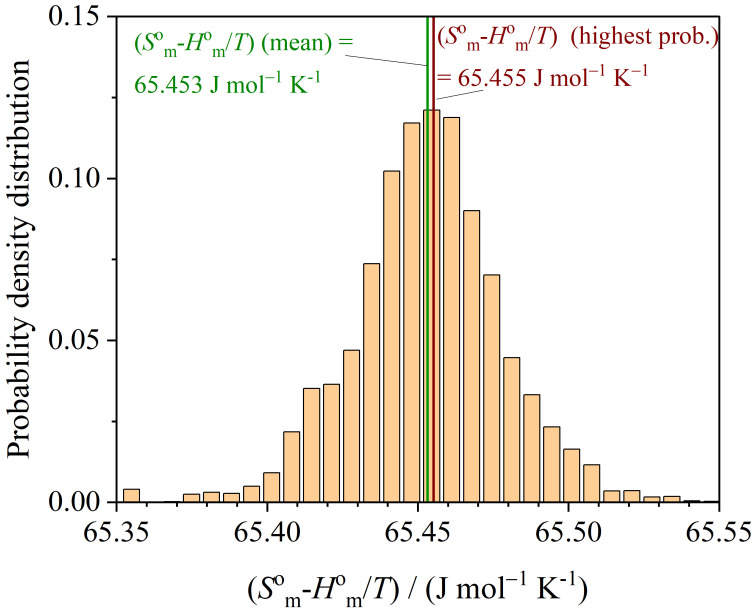
Probability density distribution of $S_{m}^{o}-H_{m}^{o}/T$ at T=298.15K.

The probability density distribution of the function $S_{m}^{o}-H_{m}^{o}/T$ at T=298.15K is presented in Figure 9.

It is worth mentioning that in some cases the highest probability lies not exactly at the position where the distribution evaluated by the “naked eye” expects the highest probability to be. This point can be explained as follows: The probability density distribution is obtained from all $4\cdot 10^{5}$ Markov chain entries. A certain entry (six parameters of the Debye–Einstein fit and the two parameters describing the function of the standard errors of the heat capacities) has the highest value of (lnP), Equation (10). This maximum probability corresponds to the minimum obtained by the Levenberg–Marquardt approach, assuming that the same function is used for the standard errors.

In summary, determining thermodynamic functions within the Bayesian framework does not pose any difficulties. The Bayesian approach allows for the calculation of thermodynamic properties as a function of temperature based on the obtained sets of fit parameters. The resulting histograms provide insights into the distribution of values of the thermodynamic functions at specific temperatures. The mean value, along with the value corresponding to the highest probability, can be determined from the histograms. Additionally, the standard errors can be estimated from the probability density distributions of the thermodynamic functions at a specific temperature.

## 4. Conclusions

For the example of ${\mathrm{SrMoO}}_{4}$, it is again demonstrated in this work that the six-parameter Debye–Einstein fit for molar heat capacities $C_{p,m}^{o}\left(T\right)$ works very well in the range 0–300 K, where ${\mathrm{SrMoO}}_{4}$ could be replaced by many crystalline solids. Two different regression methods are applied for this task; the first is based on frequentist statistics using classical least squares, the second is an application of Bayes’ theorem, numerically treated by the MCMC method. It is demonstrated that both completely different approaches not only lead to comparable results for the values of the parameters, but also to similar uncertainties of these parameters.

In addition, this investigation showcases the efficacy of the Bayesian framework to determine thermodynamic functions and their uncertainties. Based on the residuals, the parameters for the temperature-dependent function of the standard errors are optimized together with the model parameters and the Bayesian credible interval is obtained as the result.

From the correlation matrix of this example, it can be deduced that no more fitting parameters should be used in this temperature range as the correlation between these physically based parameters is partially very high. It can be seen as an advantage of the MCMC approach that the probability density distributions of the model parameters and of the derived quantities, such as the entropy *S*, enthalpy *H* and other thermodynamic functions, are revealed. Based on the results of this extented regression analysis of the molar heat capacities of ${\mathrm{SrMoO}}_{4}$, it can be recommended to use the 6p-Debye–Einstein integral approach as a standard method to fit heat capacities of many crystalline solids in the range between 0 K and 300 K.

## Figures and Tables

**Table 1:** Six-parameter Debye–Einstein fit by classical LSQ and by MCMC.

Method	*m*	$n_{1}$	$n_{2}$	$T_{D}\;/\;K$	$T_{E1}\;/\;K$	$T_{E2}\;/\;K$
LSQ	2.65±0.03	2.00±0.03	1.43±0.02	250±2	470±8	1129±29
MCMC (highest prob.)	2.624±0.020	1.985±0.030	1.439±0.003	249.5±1.0	464.3±6.0	1106.9±28.0
MCMC (mean)	2.633	1.992	1.440	249.9	467.0	1117.3

**Table 2:** The function for the standard error of the heat capacity.

Method	$s_{0}$/$J\;{\mathrm{mol}}^{-1}\;K^{-1}$	$s_{1}$/${\left(J\;{\mathrm{mol}}^{-1}\;K^{-1}\right)}^{\left(1/2\right)}$
MCMC (highest prob.)	0.029±0.007	0.007±0.002
MCMC (mean)	0.030	0.0081

**Table 3:** Correlation matrix *r* (symmetric matrix).

	*m*	$n_{1}$	$n_{2}$	$T_{D}$	$T_{E1}$	$T_{E2}$	$s_{0}$	$s_{1}$
*m*	1.0	0.31	−0.33	0.98	0.91	0.61	0.00	0.09
$n_{1}$		1.0	0.09	0.19	0.67	0.9	0.04	−0.05
$n_{2}$			1.0	−0.31	−0.26	0.21	−0.01	0.00
$T_{D}$				1.0	0.83	0.52	−0.03	0.12
$T_{E1}$					1.0	0.83	0.02	0.04
$T_{E2}$						1.0	0.02	0.01
$s_{0}$							1.0	−0.62
$s_{0}$								1.0

**Table 4:** Thermodynamic functions of ${\mathrm{SrMoO}}_{4}$ at T=298.15K derived from molar heat capacity.

Source	$S_{m}^{o}$ ($J\;{\mathrm{mol}}^{-1}\;K^{-1}$)	$H_{m}^{o}\;\left(\mathrm{kJ}\;{\mathrm{mol}}^{-1}\right)$	$S_{m}^{o}\left(T\right)-\frac{H_{m}^{o}\left(T\right)}{T}\;\left(J\;{\mathrm{mol}}^{-1}\;K^{-1}\right)$
From [26]	136.56	21.14	65.32
LM (least squares)	136.51	21.188	65.45
MCMC (highest prob.)	136.52±0.04	21.188±0.003	65.46±0.02

## Data Availability

The data presented in this study are available on request from the corresponding author.

## References

[1] Ditmars D.A., Ishihara S., Chang S.S., Bernstein G., West E.D. (1982). Enthalpy and Heat-Capacity Standard Reference Material: Synthetic Sapphire (Alpha-Al2O3) From 10 to 2250 K. J. Res. Natl. Bur. Stand..

[2] Bissengaliyeva M.R., Bekturganov N.S., Gogol D.B., Taimassova S. (2017). Low-temperature heat capacity and thermodynamic functions of natural chalcanthite. J. Chem. Thermodyn..

[3] Bissengaliyeva M.R., Knyazev A.V., Bespyatov M.A., Gogol D.B., Taimassova S.T., Zhakupov R.M., Sadyrbekov D.T. (2022). Low-temperature heat capacity and thermodynamic functions of thulium and lutetium titanates and Schottky anomaly in Tm_2_Ti_2_O_7_. J. Chem. Thermodyn..

[4] Smith A.L., Griveau J.C., Colineau E., Raison P.E., Konings R. (2015). Low temperature heat capacity of *α*-Na_2_NpO_4_. Thermochim. Acta.

[5] Kelley K.K., King E.G. (1961). Contributions to the Data on Theoretical Metallurgy. XIV. Entropies of the Elements and Inorganic Compounds.

[6] Chen Q., Sundman B. (2001). Modeling of thermodynamic properties for Bcc, Fcc, liquid, and amorphous iron. J. Phase. Equilib..

[7] Musikhin A., Naumov V., Bespyatov M., Ivannikova N. (2015). The heat capacity of Li_2_MoO_4_ in the temperature range 6–310 K. J. Alloys Compd..

[8] Roslyakova I., Sundman B., Dette H., Zhang L., Steinbach I. (2016). Modeling of Gibbs energies of pure elements down to 0 K using segmented regression. Calphad.

[9] Gamsjäger E., Morishita M., Gamsjäger H. (2016). Calculating entropies of alkaline earth metal molybdates. Monatshefte für Chemie Chem. Mon..

[10] Wu L., Schliesser J.M., Woodfield B.F., Xu H., Navrotsky A. (2016). Heat capacities, standard entropies and Gibbs energies of Sr-, Rb- and Cs-substituted barium aluminotitanate hollandites. J. Chem. Thermodyn..

[11] Morishita M., Kinoshita Y., Houshiyama H., Nozaki A., Yamamoto H. (2017). Thermodynamic properties for calcium molybdate, molybdenum tri-oxide and aqueous molybdate ion. J. Chem. Thermodyn..

[12] Gamsjäger E., Wiessner M. (2018). Low temperature heat capacities and thermodynamic functions described by Debye-Einstein integrals. Monatshefte für Chemie Chem. Mon..

[13] Shumway S.G., Wilson J., Lilova K., Subramani T., Navrotsky A., Woodfield B.F. (2022). The low-temperature heat capacity and thermodynamic properties of greigite (Fe_3_S_4_). J. Chem. Thermodyn..

[14] Gabriel J.J., Paulson N.H., Duong T.C., Becker C.A., Tavazza F., Kattner U.R., Stan M. (2021). Bayesian automated weighting of aggregated DFT, MD, and experimental data for candidate thermodynamic models of aluminum with uncertainty quantification. Materialia.

[15] Honarmandi P., Duong T.C., Ghoreishi S.F., Allaire D., Arroyave R. (2019). Bayesian uncertainty quantification and information fusion in CALPHAD-based thermodynamic modeling. Acta Mater..

[16] Paulson N.H., Bocklund B.J., Otis R.A., Liu Z.K., Stan M. (2019). Quantified uncertainty in thermodynamic modeling for materials design. Acta Mater..

[17] Malakhov D.V. (1997). Confidence intervals of calculated phase boundaries. Calphad.

[18] Königsberger E., Gamsjäger H. (1990). Analysis of phase diagrams employing Bayesian excess parameter estimation. Monatshefte für Chemie Chem. Mon..

[19] Vrugt J.A., Ter Braak C.J.F. (2011). DREAM_(D): An adaptive Markov Chain Monte Carlo simulation algorithm to solve discrete, noncontinuous, and combinatorial posterior parameter estimation problems. Hydrol. Earth Syst. Sci..

[20] Gagin A., Levin I. (2015). Accounting for unknown systematic errors in Rietveld refinements: A Bayesian statistics approach. J. Appl. Crystallogr..

[21] Box G.E.P. (1976). Science and Statistics. J. Am. Stat. Assoc..

[22] Boerio-Goates J., Stevens R., Hom B.K., Woodfield B.F., Piccione P.M., Davis M.E., Navrotsky A. (2002). Heat capacities, third-law entropies and thermodynamic functions of *SiO*_2_ molecular sieves from T = 0 K to 400 K. J. Chem. Thermodyn..

[23] Dachs E., Benisek A. (2011). A sample-saving method for heat capacity measurements on powders using relaxation calorimetry. Cryogenics.

[24] Bissengaliyeva M.R., Gogol D.B., Taimassova S., Bekturganov N.S. (2012). Experimental determination of thermodynamic characteristics of smithsonite. J. Chem. Thermodyn..

[25] Bissengaliyeva M.R., Gogol D.B., Taimassova S.T., Bekturganov N.S. (2012). The heat capacity and thermodynamic functions of cerussite. J. Chem. Thermodyn..

[26] Morishita M., Houshiyama H. (2015). The Third Law Entropy of Strontium Molybdates. Mater. Trans..

[27] Quantum Design (2004). Quantum Design: Physical Property Measurement System: Heat Capacity Option User’s Manual.

[28] Dachs E., Bertoldi C. (2005). Precision and accuracy of the heat-pulse calorimetric technique: Lowtemperature heat capacities of milligram-sized synthetic mineral samples. Eur. J. Mineral..

[29] Ogris D.M., Gamsjäger E. (2021). Heat capacities and standard entropies and enthalpies of some compounds essential for steelmaking and refractory design approximated by Debye-Einstein integrals. Calphad.

[30] Sivia D.S. (2006). Data Analysis: A Bayesian Tutorial; for Scientists and Engineers.

[31] Braak C.J.F.T. (2006). A Markov Chain Monte Carlo version of the genetic algorithm Differential Evolution: Easy Bayesian computing for real parameter spaces. Stat. Comput..

[32] Wiessner M., Angerer P., van der Zwaag S., Gamsjäger E. (2021). Transient phase fraction and dislocation density estimation from in-situ X-ray diffraction data with a low signal-to-noise ratio using a Bayesian approach to the Rietveld analysis. Mater. Charact..

[33] OriginLab Corporation (2022). OriginPro, Version 2022b.

